# BECLIN-1-Mediated Autophagy Suppresses Silica Nanoparticle-Induced Testicular Toxicity via the Inhibition of Caspase 8-Mediated Cell Apoptosis in Leydig Cells

**DOI:** 10.3390/cells11121863

**Published:** 2022-06-07

**Authors:** Qianru Zhang, Jason William Grunberger, Nitish Khurana, Xin Zhou, Xianyu Xu, Hamidreza Ghandehari, Fenglei Chen

**Affiliations:** 1 Institute of Comparative Medicine, College of Veterinary Medicine, Yangzhou University, Yangzhou 225009, China; longlongzhang12345@126.com (Q.Z.); zhou_xin@126.com (X.Z.); xuxianyu@yzu.edu.cn (X.X.); 2 Jiangsu Co-innovation Center for Prevention and Control of Important Animal Infectious Diseases and Zoonoses, Yangzhou University, Yangzhou 225009, China; 3 Joint International Research Laboratory of Agriculture and Agri-Product Safety, The Ministry of Education of China, Yangzhou University, Yangzhou 225009, China; 4 Department of Pharmaceutics and Pharmaceutical Chemistry, University of Utah, Salt Lake City, UT 84112-5001, USA; u1204188@utah.edu (J.W.G.); nitish.khurana@utah.edu (N.K.); hamid.ghandehari@utah.edu (H.G.); 5 Utah Center for Nanomedicine, University of Utah, Salt Lake City, UT 84112-5001, USA; 6 Department of Biomedical Engineering, University of Utah, Salt Lake City, UT 84112-5001, USA

**Keywords:** silica nanoparticles, BECLIN-1, autophagy, apoptosis, Leydig cells, testicular toxicity

## Abstract

Accumulation of silica nanoparticles (SNPs) in the testes leads to male reproductive toxicity. However, little is known about the effect and mechanistic insights of SNP-induced autophagy on apoptosis in Leydig cells. In this study, we aimed to verify the role of SNP-induced autophagy in apoptosis and explore the possible underlying mechanism in mouse primary Leydig cells (PLCs). H&E staining showed that SNPs changed the histological structures of the testes, including a reduction in the Leydig cell populations in vivo. CCK-8 assay showed that SNPs decreased cell viability, and flow cytometry showed that SNPs increased cell apoptosis, both in a dose-dependent manner in vitro. Additionally, Western blotting further found that SNPs activated autophagy by an increase in BECLIN-1, ATG16L, and LC3-II levels and promoted the intrinsic pathway of apoptosis by an increase in the BAX/BCL-2 ratio, cleaved the caspase 8 and caspase 3 levels. Furthermore, autophagy decreased SNP-induced apoptosis via regulation of the caspase 8 level combined with rapamycin, 3-methyladenine, and chloroquine. BECLIN-1 depletion increased the caspase 8 level, leading to an increase in SNP-induced cell apoptosis. Collectively, this evidence demonstrates that SNPs activated BECLIN-1-mediated autophagy, which prevented SNP-induced testicular toxicity via the inhibition of caspase 8-mediated cell apoptosis in Leydig cells.

## 1. Introduction

Silica nanoparticles (SNPs), one of the readily available nanomaterials, have been widely used in agriculture, cosmetics, food additives, and biomedical applications [1]. Owing to their wide applicability, especially in consumer products and as an additive in biomedical formulations, the risk of human and animal exposure needs to be investigated [2,3]. Depending on the physicochemical properties and duration and route of exposure, SNPs can potentially cause toxicity in a variety of organs, such as liver, kidneys, brain, lungs, ovaries, and testes [4,5,6]. For example, SNPs can cross the blood–testis barrier (BTB) and distribute in testicular tissue, resulting in testicular toxicity [6,7,8]. 

It is well-known that testes are essential organs in the male reproductive system, whose main functions are to produce sperms and secrete androgen. Previous studies found that they are vulnerable and sensitive to nanoparticle (NP) accumulation [9], such as zinc oxide nanoparticles (ZnONPs) [10], cerium oxide nanoparticles (CeO2 NPs) [11], and SNPs [7]. Testicular toxicity results from the accumulation of NPs through different routes including blood circulation or direct contact with NPs. SNPs are known to cause seminiferous tubule damage, resulting in a decrease in motility of sperm [6]. However, the mechanisms of SNP-induced testicular toxicity are not well-understood. Studies have reported the toxicity of SNPs in spermatogenesis; however, the effect of SNPs on Leydig cells is rarely discussed. Leydig cells play an essential role in promoting spermatogenesis as they help in testosterone synthesis and secretion [12]. The testicular toxicity caused by SNPs via the induction of apoptosis in Leydig cells needs further investigation.

Autophagy is a protective mechanism to maintain cellular homeostasis [13]. The steps of the autophagic pathway include initiation, nucleation, elongation of the phagophore, maturation of the autophagosome, and formation of the autolysosome [14,15]. BECLIN-1 combined with vacuolar protein sorting (VPS)-34 and -15 regulates the nucleation stage of autophagy [16]. BECLIN-1, an ortholog of the ATG6/VPS30 protein in yeast, is a B cell lymphoma-2 (BCL-2) homology (BH)-3 domain-only protein [17]. BCL-2 can interact with the BH3 domain of BECLIN-1, resulting in inhibition of autophagy activation. Both autophagy blockage and autophagy genes depletion can cause cell apoptosis under stress stimulation [18]. Caspases, a family of cysteine aspartyl proteases, have an important role in inducing apoptosis. Caspase -8 is a death receptor effector and can be degraded by autophagy [19]. Additionally, BECLIN-1 can interact with caspase 8 to inhibit death receptor-mediated cell apoptosis [20]. Caspase 8 also combines with Bid, which can be cleaved by caspase 8 and translocated to the mitochondria, to cooperate in mitochondria-mediated cell apoptosis [21]. These results indicate the crosstalk between apoptosis and autophagy.

Studies have shown that autophagy occurs in the toxicity induced by SNPs. For instance, autophagy prevents human lung fibroblast (MRC-5) cells and RAW 264.7 macrophage cells against cytotoxicity of 156 nm (hydrodynamic sizes in ultrapure water) amorphous and 236 nm (hydrodynamic sizes in water) nonporous spherical SNPs, respectively [22,23]. The activation of autophagy attenuates 150 nm amorphous spherical SNP-induced inflammation in RAW 264.7 macrophage cells [24]. Meanwhile, 105 nm (hydrodynamic sizes in ultrapure water) nonporous spherical SNP-induced autophagy triggered autophagic cell death in human hepatocellular carcinoma (HepG2) cells [25]. However, whether autophagy is related to apoptosis in SNP-induced testicular toxicity has not been investigated. To this end, this study aimed to determine the effect of SNP-activated autophagy on mouse Leydig cell apoptosis and the possible mechanisms.

## 2. Materials and Methods

### 2.1. Reagents

Rabbit anti-cleaved caspase 3 antibody (9664) was purchased from Cell Signaling Technology (Danvers, MA, USA). Rabbit anti-BCL-2 antibody (ab182858), rabbit anti-BAX antibody (ab32503), rabbit anti-cleaved caspase 8 antibody (ab25901), rabbit anti-ATG5 antibody (ab108327), rabbit anti-ATG16L antibody (ab187671), and rabbit anti-ATG4B antibody (ab154843) were purchased from Abcam Ltd. (Cambridge, MA, USA). Rabbit anti-β-actin antibody (AC026) and rabbit anti-P62 antibody (A19700) were purchased from ABclonal Inc. (Wuhan, China). Rabbit anti-LC3 antibody (L7543), 3-methyladenine (3-MA; M9281), chloroquine (CQ; C6628), collagenase I (C5138), and rapamycin (Rap; V900930) were purchased from Sigma Aldrich Chemical Co. (St. Louis, MO, USA). 

### 2.2. SNP Preparation and Characterization

According to the Stöber method, the synthesis of SNPs was described in our previous study [16]. Briefly, absolute ethanol (1700 mmol), distilled water (155 mmol), and ammonium hydroxide (26 mmol) were mixed in a flask with a stirring rate of 400 rpm for 10 min. Next, TEOS (16 mmol) was added dropwise, and the reaction was left under stirring for 24 h at room temperature. The synthesized SNPs were pelleted and precipitated by centrifugation using an Avanti J-15R centrifuge (Beckman Coulter Inc., Indianapolis, IN, USA) with a stirring rate of 15,000 rpm for 20 min, washed with distilled water and 95% ethanol three times, and stored in absolute ethanol at a concentration of 4 mg/mL. Before use, the stock solution was recentrifuged at 18,000 rpm for 30 min and redispersed in sterilized water. The physicochemical characteristics of SNPs were analyzed by transmission electron microscopy (TEM; Tecnai 12; Royal Philips, Amsterdam, The Netherlands) and a Zetasizer Nano ZS (Malvern Instruments Ltd., Worcestershire, UK).

### 2.3. Animals and Treatment with SNPs

Male C57BL/6 mice (8-week-old, 20.0 ± 2.0 g) were obtained from the Comparative Medicine Center of Yangzhou University and maintained under standard conditions at the temperature range of 23–25 °C, 55–60% humidity. and a 12 h light/dark cycle. 

The mice were provided with about 5 g of food and unlimited purified water and were randomly divided into a control group and an SNP treatment group. On the first day, the control group was administered with 0.2 mL of saline via tail vein injection (*n* = 6). The SNP treatment group was administered with 25 mg/kg of SNPs via tail vein injection (*n* = 6) as described previously [4]. After injection, the mice were weighed and sacrificed after 7 days. The testes were removed from the abdominal cavities and immersed in a modified Davidson’s fluid tissue fixative (including 95% alcohol, formaldehyde, glacial acetic acid, and ultrapure water in a ratio of 3:2:1:2) for hematoxylin-eosin (H&E) staining.

### 2.4. H&E Staining

The fixed testis samples were dehydrated by graded ethanol (50%, 75%, 80%, 95%, and absolute ethanol for 1 h each), placed in xylene until transparent, and then embedded in paraffin; 5 µm thick sections were cut continuously for subsequent staining. The sections were deparaffinized in xylene and hydrated with graded ethanol (absolute ethanol I and II for 5 min, respectively; 95% alcohol I and II for 3 min, respectively; 80% and 70% for 3 min). Next, the tissue sections were stained with hematoxylin for 3 min, washed with water, and stained in eosin for 12 s. After cleaning with xylene and drying, the sections were mounted with neutral balsam. The morphological structure was observed by digital microscopy (BA400, Motic, Amoy, Xiamen, China). 

### 2.5. PLC Culture In Vitro and Treatment with SNPs

The testes were removed from 8-week-old male mice and collected in DMEM/F12. After decapsulation, they were added to DMEM/F12 containing 1 mg/mL of collagenase I to separate PLCs from other testicular cells and incubated in a shaking water bath at 37 °C for 10 min. After removing the floc of the testes, the same amount of DMEM/F12 with 10% FBS was added to stop digestion. After filtration, the PLCs were distributed in a culture medium inside 60 mm tissue culture dishes containing DMEM/F12 with 10% FBS and 100 mg/L of penicillin/streptomycin at 37 °C and 5% CO_2_. After 6 h, the medium was removed and replaced with fresh DMEM/F12. When cell confluency reached 70–80%, the PLCs were treated with SNPs. 

### 2.6. Determination of SNP Uptake

The SNP-treated PLCs were carefully washed in 0.01 M PBS (pH = 7.4), fixed in 4% paraformaldehyde and 2.5% glutaraldehyde in PBS for 24 h. The PLCs were washed with PBS three times, embedded in agarose gel (2%), postfixed in osmium tetroxide (4%), and washed again with PBS. Next, the samples were dehydrated with gradient concentrations of ethanol (30%, 50%, 70%, 80%, 90%, and 100%) and embedded in epoxy resin. The sections were cut using an ultramicrotome and stained with uranyl acetate–lead citrate (3%). Finally, the ultrastructure of the PLCs was observed by TEM.

### 2.7. Cell Viability Assay

Cell viability of the PLCs was assessed after SNP treatment using Cell Counting Kit 8 (CCK-8, New Cell and Molecular Biotech Co. Ltd., Suzhou, China); 3 × 10^4^ cells/200 µL medium/well were seeded in 96-well plates for 24 h and then treated with various doses of SNPs (control, 100, 200, 400, 600, 800, 1000, and 1200 μg/mL) for 24 h; 10 μL of CCK-8 were added into each well and incubated for 2 h at 37 °C. Finally, the absorbance readings were taken at 450 nm using an enzyme-linked immunosorbent assay (ELISA) plate reader (Model 680, Bio-Rad, and Hercules, CA, USA).

### 2.8. Cell Apoptosis Assay

Cell apoptosis in the PLCs was determined after SNP treatment by flow cytometry (EPICS Altra, Beckman Coulter Cytomics Altra, Brea, CA, USA); 2 × 10^6^ PLCs/well were seeded into a 6-well culture plate for 24 h and then treated with various concentrations of SNPs (control, 200, 400, and 800 µg/mL) for 24 h. The cells were washed twice with PBS, trypsinized, centrifuged, and harvested. After resuspension in 500 μL of a binding buffer, 5 μL of Annexin V-FITC or V-PE and propidium iodide (PI) (Nanjing KeyGen Biotech, Nanjing, China) were added into the cells. The cell apoptotic rate was determined within 1 h by flow cytometry.

### 2.9. Construction of Recombinant BECLIN-1 shRNA and Cell Transduction 

Two BECLIN-1 shRNA vectors (shBecl1-1 and shBecl1-2) and a negative control shRNA vector (shNC) were preserved in our laboratory [26]. The recombinant BECLIN-1 lentiviruses were packaged using HEK 293T cells as described previously [27]. Lentiviral particles (multiplicity of infection (MOI) = 20) were transduced into the PLCs. After 48 h, the cells were treated with SNPs. Finally, the cells were harvested for further experiments.

### 2.10. Monodansylcadaverine (MDC) Staining Detection

MDC staining was performed to monitor the activation of autophagy in the PLCs after SNP treatment as described previously [26]. Briefly, the PLCs were cultured on sterile cover slips and then treated with various doses of SNPs for 12 h. Next, the PLCs were incubated with MDC solution in the dark. Finally, the PLCs were examined by laser scanning confocal microscopy (TCS SP8 STED; Wetzlar, Hessen, Germany).

### 2.11. Western Blotting

The PLCs were harvested for protein extraction using a whole cell lysis kit (Nanjing KeyGen Biotech, Nanjing, China). The total protein concentration of the samples was determined with a BCA protein assay kit (Nanjing KeyGen Biotech, Nanjing, China) according to the manufacturer’s instructions. Equal amounts of protein (20 μg) were separated with 10% SDS-PAGE gels and transferred to PVDF membranes by electroblotting. The transferred membranes were blocked with 10% nonfat milk for 1 h at room temperature and then incubated overnight at 4 °C with the following primary antibodies: BCL-2 (1:1000), BAX (1:1000), ATG5 (1:1000), ATG16L (1:1000), ATG4B (1:1000), P62 antibody (1:500), LC3-II (1:1000), and β-actin (1:5000). Next, the membranes were incubated with HRP-linked goat anti-mouse or goat anti-rabbit IgG (1:5000 dilutions) for 1 h at room temperature. The signal was measured using an enhanced chemiluminescence (ECL) kit (New Cell and Molecular Biotech Co. Ltd., Suzhou, China), and the protein band intensity was quantified using Quantity One software (Bio-Rad, Hercules, CA, USA).

### 2.12. Statistical Analysis

All the statistical analyses were performed using the Statistical Package for the Social Sciences (SPSS) software (version 18.0; SPSS, Chicago, IL, USA). An independent-samples *t*-test was used to compare two groups. One-way ANOVA was used to analyze more than two groups followed by the least significant difference (LSD) test to compare various groups. All the values are presented as the means ± SEM from independent experiments performed in triplicate, and the difference was statistically at *p* < 0.05.

## 3. Results

### 3.1. Characterization of SNPs

**Figure 1:**
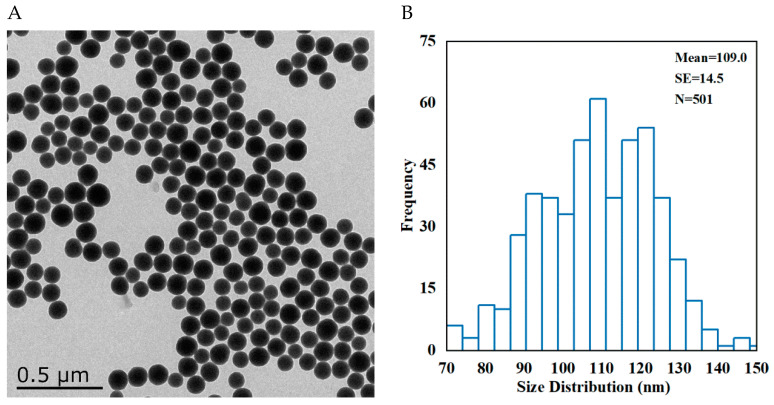
The morphology and size of SNPs. (**A**) Representative TEM image of spherical SNPs. Scale bar, 0.5 μm. (**B**) Distribution diagram of SNPs in (**A**).

The morphology of the Stöber SNPs was determined by TEM. Low-magnification TEM images showed the SNP particles to be of spherical shape with low polydispersity (Figure 1A). The average diameter of SNPs was 109.0 ± 14.5 nm which was determined by randomly counting and measuring more than 500 particles (Figure 1B). The hydrodynamic diameter of SNPs and the zeta potential of SNPs can be obtained from our previous study [26].

### 3.2. SNPs Change the Histological Structures of Testes In Vivo

**Figure 2:**
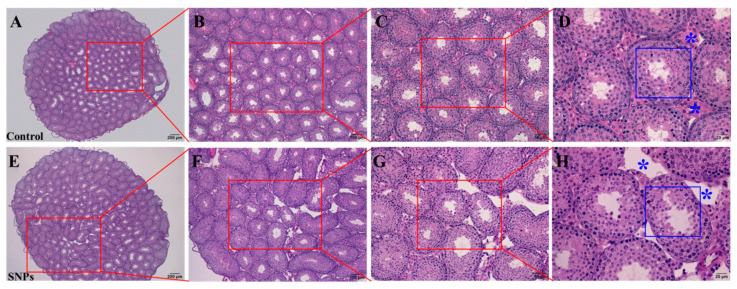
Effects of SNPs on the histological structures of the testes in vivo. (**A**) The image of the testes in the control group by H&E staining. (**B**) The selected red area in (**A**). (**C**) The selected red area in **B**. (**D**) The selected red area in (**C**). (**E**) The image of the testes in the SNP-treated group by H&E staining. (**F**) The selected red area in (**E**). (**G**) The selected red area in (**F**). (**H**) The selected red area in **G**. The red arrow represents the spermatogenic cells of the seminiferous tubules; the blue asterisk represents Leydig cells. Scale bars, 200 μm (**A**,**E**), 100 μm (**B**,**F**), 50 μm (**C**,**G**), and 20 μm (**D**,**H**).

To observe the toxic effects of SNPs on testes in vivo, the histological structures of the testes were observed by H&E staining. The results showed that the testes from the control group had normal testicular architecture and germinal cell arrangement (Figure 2A–D). The Leydig cells and interstitial tissue had the same size and their count was normal (Figure 2D). All the spermatogenic cells and Sertoli cells were normal (Figure 2D). However, the testes from the SNP group showed marked atrophy and deformation of the seminiferous tubules characterized by a reduction in the Leydig cell populations and disorganization of spermatogenic cell layers (Figure 2E–H). 

### 3.3. SNP Cellular Uptake in Primary Leydig Cells (PLCs) 

**Figure 3:**
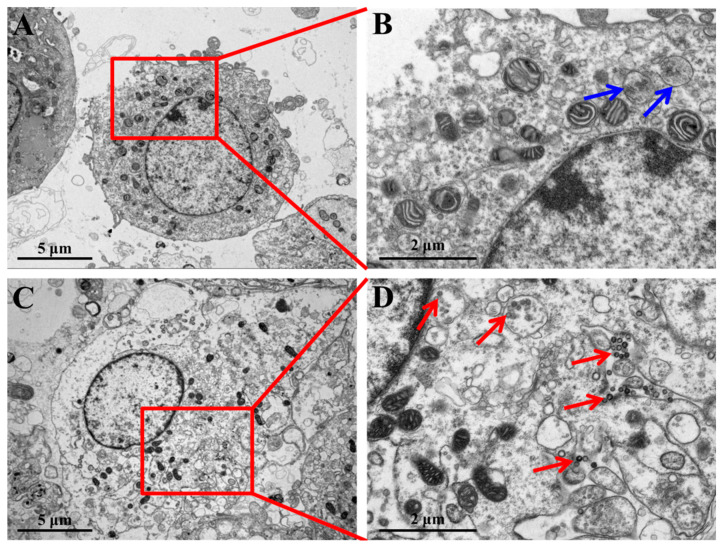
TEM images of cellular uptake of SNPs. (**A**) PLCs treated with 400 µg/mL SNPs for 12 h. (**B**) The selected red square area in (**A**). (**C**) Control PLCs. (**D**) The selected red square area in (**C**). The red arrows indicate the vesicles where SNPs were enclosed. The blue arrows indicate the vesicles where SNPs were not enclosed. Scale bar, 5 µm (**A**,**C**) and 2 µm (**B**,**D**).

To determine whether SNPs can be taken up by PLCs, the localization of SNPs within the PLCs was monitored by TEM. The results showed that there were no particles in the control group (Figure 3A,B), while the accumulation of SNPs was mainly localized in vesicles of the cytoplasm in the PLCs in the SNP group (Figure 3C,D).

### 3.4. SNPs Decreased Cell Viability and Increased Cell Apoptosis in the PLCs

**Figure 4:**
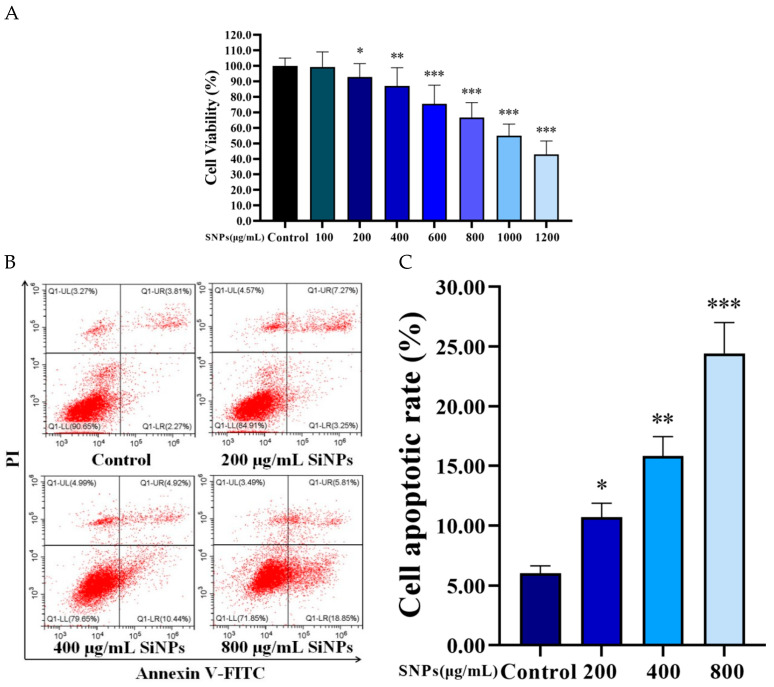
Effect on the viability and apoptosis after SNP treatment. (**A**) Cell viability was determined by CCK-8. The PLCs were treated with control, 100, 200, 400, 600, 800, 1000, and 1200 μg/mL SNPs for 24 h. (**B**) The apoptotic rate was detected by flow cytometry. The PLCs were treated with control, 200, 400, and 800 μg/mL SNPs for 24 h. (**C**) Quantification and statistical analysis of the apoptotic rate. Note: * *p* < 0.05, ** *p* < 0.01, and *** *p* < 0.001 compared to the control.

To determine the effect of SNPs on cell viability and apoptosis, CCK-8 and flow cytometry were employed to analyze the samples after different doses of SNP treatment for 24 h. The CCK-8 assay showed that SNPs (above 200 μg/mL) decreased cell viability in a dose-dependent manner (Figure 4A). The flow cytometry assay showed that SNPs increased cell apoptosis in a dose-dependent manner (Figure 4B,C). The apoptotic rate was significantly increased in the groups after 200, 400, and 800 μg/mL SNP treatments (10.70 ± 1.17%, 15.83 ± 1.63%, and 24.42 ± 2.58%, respectively) compared to the control group (6.01 ± 0.63%) (Figure 4B,C).

### 3.5. SNPs Activated Autophagy and the Apoptotic Pathway in the PLCs

**Figure 5:**
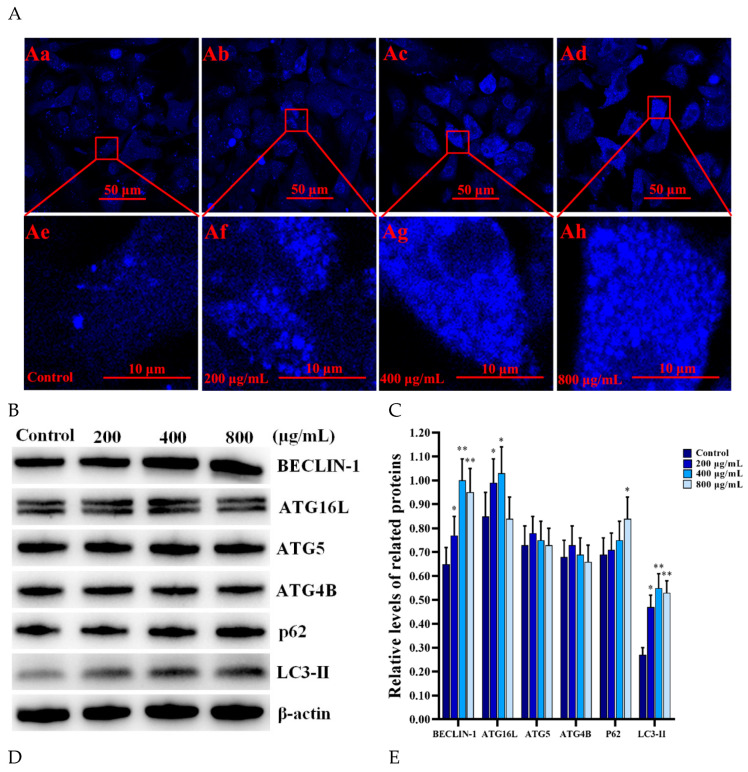

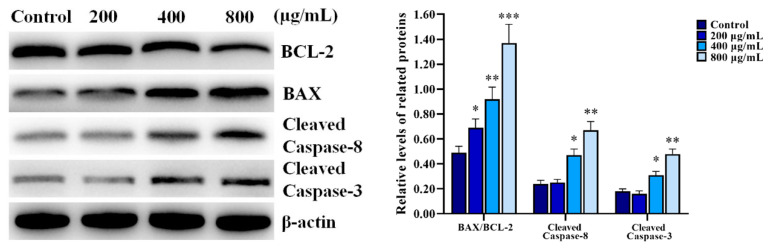
Effect of SNPs on the activation of autophagy and the apoptotic pathway. (**A**) Autophagy was determined by MDC staining. Scale bar, 50 μm (**Aa**–**Ad**) and 10 μm (**Ae**–**Ah**). (**B**) Autophagy-related proteins were detected by Western blotting. (**C**) Bar graphs of the analyses of band intensity in (**B**) as the relative ratio of the proteins related to β-actin. (**D**) Apoptosis-related proteins were detected by Western blotting. The PLCs were treated with control, 200, 400, and 800 μg/mL SNPs for 12 h. (**E**) Bar graphs of the statistical analyses of band intensity in (**D**) as the relative ratio of the proteins related to β-actin. Note: * *p* < 0.05, ** *p* < 0.01, and *** *p* < 0.001 compared to the control.

To ensure whether SNPs activate autophagy and the intrinsic pathway of apoptosis, MDC staining and Western blotting were employed to analyze the samples after different doses of SNP treatment for 12 h. MDC staining showed that SNPs enhanced the accumulation of autophagic vacuoles in the PLCs in a dose-dependent manner (Figure 5A). The fluorescent intensity was significantly increased in the groups after 200, 400, and 800 μg/mL SNP treatments compared to the control group (Figure 5A). Furthermore, Western blotting showed that the levels of ATG16L, BECLIN-1, LC3-II, the BAX/BCL-2 ratio, cleaved caspase 8, and cleaved caspase 3 were significantly increased in a certain dose-dependent manner (Figure 5B–E). The P62 level was significantly increased in the group after 800 μg/mL SNP treatment and had no significant difference between the groups after 200 and 400 μg/mL SNP treatments compared to the control group (Figure 5B,C). The ATG5 and ATG4B levels had no significant difference between the SNP and control groups (Figure 5B,C). The BAX/BCL-2 ratio was significantly increased in a dose-dependent manner; cleaved caspase 8 and cleaved caspase 3 were also significantly increased in the groups after 400 and 800 μg/mL SNP treatments and had no significant difference between the groups after 200 μg/mL SNP treatments compared to the control group (Figure 5D,E) 

### 3.6. Autophagy Inhibited SNP-Induced Apoptosis in the PLCs

**Figure 6:**
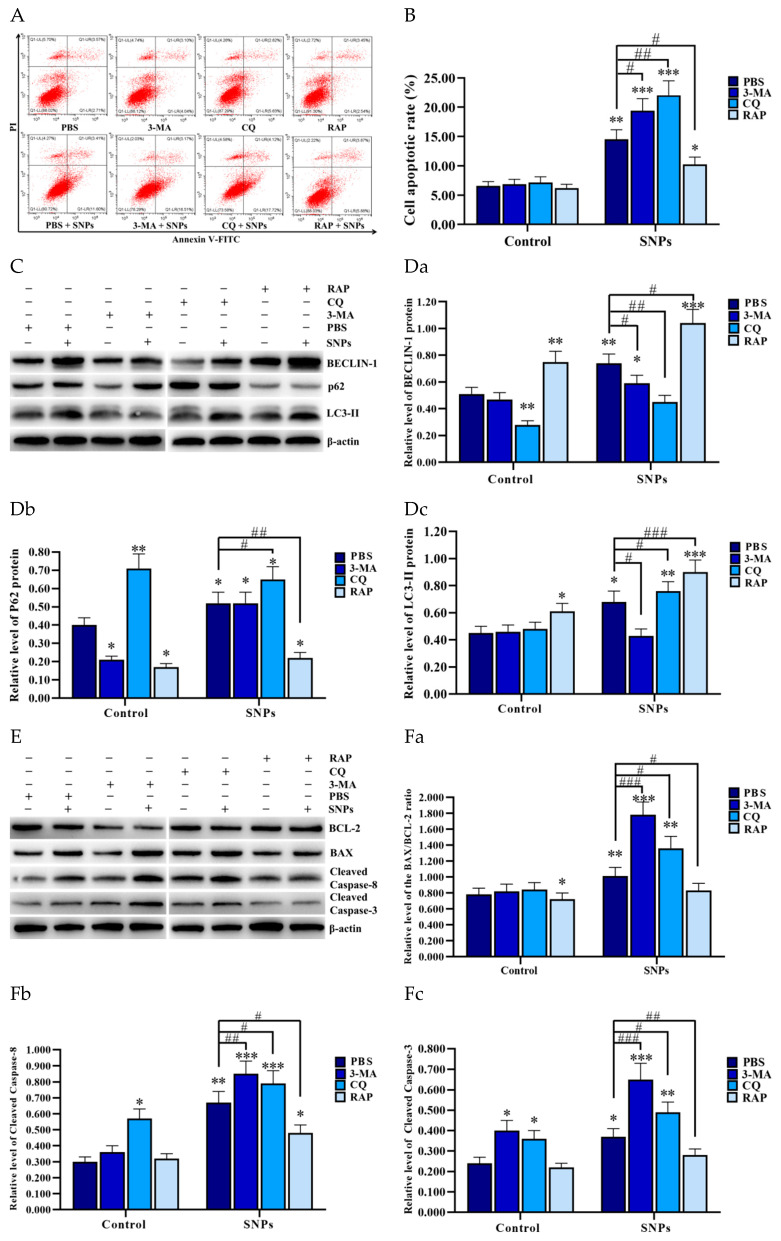
Effect of autophagy on SNP-induced apoptosis. (**A**) The apoptotic rate was detected by flow cytometry. The PLCs were treated with 400 μg/mL SNPs for 24 h pretreatment with 3-MA (0.2 µM), CQ (5 µM), and RAP (1 µM) for 6 h. (**B**) Bar graphs of the statistical analysis of the apoptotic rate in (**A**). (**C**) Autophagy-related proteins were detected by Western blotting. (**D**) Bar graphs of the statistical analyses of band intensity in **C** as the relative ratio of BECLIN-1 (**Da**), P62 (**Db**), and LC3-II (**Dc**), respectively, to β-actin. (**E**) Apoptosis-related proteins were detected by Western blotting. The PLCs were treated with 400 μg/mL SNPs for 12 h pretreatment with 3-MA (0.2 µM), CQ (5 µM), and RAP (1 µM) for 6 h. (**F**) Bar graphs of the statistical analyses of band intensity in (**E**) as the relative ratio of the BAX/BCL-2 ratio (**Fa**), cleaved caspase 8 (**Fb**), and cleaved caspase 3 (**Fc**), respectively, to β-actin. Note: * *p* < 0.05, ** *p* < 0.01, and *** *p* < 0.001 compared to the PBS group; # *p* < 0.05, ## *p* < 0.01, and ### *p* < 0.001 compared to the PBS + SNP group.

To investigate the effect of autophagy on SNP-induced apoptosis, flow cytometry and Western blotting were employed to analyze the samples after SNP treatment combined with PBS (control), early autophagy inhibitor 3-MA, late inhibitor CQ, and activator Rap. The flow cytometry assay showed that the activation of autophagy decreased SNP-induced apoptosis, whereas the inhibition of autophagy increased SNP-induced apoptosis in the PLCs (Figure 6A,B). The apoptotic rate was increased in the 3-MA + SNP (19.38 ± 2.08%) and the CQ + SNP (22.04 ± 2.46%) groups, whereas it was decreased in the RAP + SNP group (10.25 ± 1.24%) compared to the PBS + SNP group (14.51 ± 1.65%) (Figure 6A,B). Western blotting showed that pretreatment with 3-MA decreased the BECLIN-1 and LC3-II levels, whereas it increased the BAX/BCL-2 ratio and the cleaved caspase 8 and cleaved caspase 3 levels compared to the PBS + SNP group (Figure 6C–Fc). Pretreatment with CQ decreased the BECLIN-1 level, whereas it increased P62, LC3-II, the BAX/BCL-2 ratio, and the cleaved caspase 8 and cleaved caspase 3 levels compared to the PBS + SNP group (Figure 6C–Fc). Pretreatment with RAP increased the BECLIN-1 and LC3-II levels, whereas it decreased P62, the BAX/BCL-2 ratio, and the cleaved caspase 8 and cleaved caspase 3 levels compared to the PBS + SNP group (Figure 6C–Fc).

### 3.7. BECLIN-1 Depletion Increased SNP-Induced Apoptosis in the PLCs

**Figure 7:**
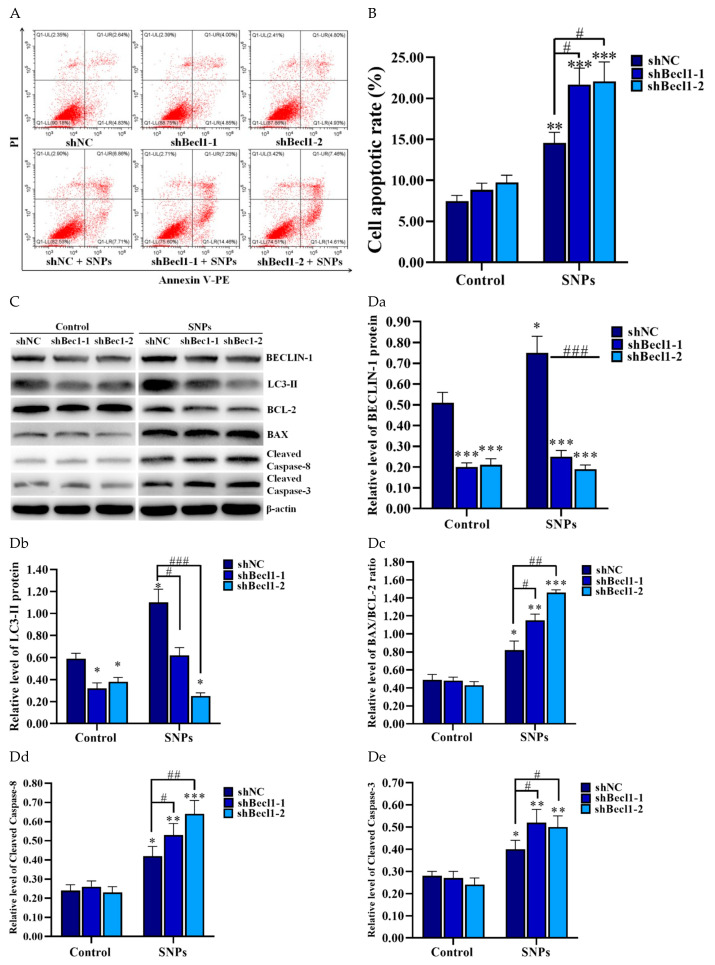
Effect of BECLIN-1 depletion on SNP-induced apoptosis. (**A**) The apoptotic rate was detected by flow cytometry. The PLCs were transduced with BECLIN-1 shRNA lentivirus for 48 h and then treated with 400 μg/mL SNPs for 24 h. (**B**) Bar graphs of the statistical analysis of the apoptotic rate in (**A**). (**C**) Autophagy and apoptosis-related proteins were detected by Western blotting. The PLCs were transduced with BECLIN-1 shRNA lentivirus for 48 h and then treated with 400 μg/mL SNPs for 12 h. (**D**) Bar graphs of the protein band intensity in (**C**) were analyzed as the relative ratio of BECLIN-1 (**Da**), LC3-II (**Db**), the BAX/BCL-2 ratio (**Dc**), cleaved caspase 8 (**Dd**), and cleaved caspase 3 (**De**), respectively, to β-actin. Note: * *p* < 0.05, ** *p* < 0.01, and *** *p* < 0.001 compared to the shNC group; # *p* < 0.05, ## *p* < 0.01, and ### p < 0.001 compared to the shNC + SNP group.

To further determine the effect of autophagy on SNP-induced apoptosis, flow cytometry and Western blotting were employed to analyze the samples after shBecl1-1, shBecl1-2, and shNC lentivirus transduction and further SNP treatment. The flow cytometry assay showed that knockdown of BECLIN-1 increased SNP-induced apoptosis in the PLCs (Figure 7A,B). The apoptotic rate was significantly increased in the shBec1-1 + SNP (21.69 ± 2.00%) and the shBec1-2 + SNP (22.07 ± 2.40%) groups compared to the shNC + SNP group (14.57 ± 1.30%) (Figure 7A,B). Western blotting showed that the knockdown of BECLIN-1 significantly decreased the BECLIN-1 and LC3-II levels (Figure 7C, Da and Db), whereas it increased the BAX/BCL-2 ratio and the cleaved caspase 8 and cleaved caspase 3 levels compared to the shNC + SNP group (Figure 7C, Dc–De).

## 4. Discussion

Numerous studies demonstrate that SNPs can cause toxicity in the male reproductive system [28,29,30,31]. SNPs can accumulate in testes, damage seminiferous tubules and Leydig cells, and inhibit spermatogenesis, in turn resulting in a decrease in the quality and quantity of sperms via intravenous [8], intramuscular [32], or inhalation [6] exposure routes. Leydig cells play an essential role in promoting spermatogenesis as they help in testosterone synthesis and secretion. However, the mechanisms of Leydig cell damage have not been studied. In this study, we demonstrated that SNPs caused cell apoptosis in mouse Leydig cells and activated BECLIN-1-mediated autophagy. Furthermore, BECLIN-1-mediated autophagy prevented SNP-induced cytotoxicity via the inhibition of the mitochondria-mediated apoptotic pathway and caspase-8-mediated death signaling, which provided evidence that autophagy and apoptosis in Leydig cells result in SNP-induced toxicity.

Autophagy is a conserved quality control pathway that can clear damaged organelles and degrade misfolded or aggregated proteins, as well as remove intracellular foreign bodies to restore the cellular environmental homeostasis [33]. Autophagy is an evolutionarily conserved process that is carried out by numerous autophagy-related (ATG) proteins [33,34]. BECLIN-1, combined with the ATG1/UNC51-like kinase (ULK) complex and the VPS34–VPS15 complex, aids in autophagosome formation at the initiation of autophagy [35]. ATG16L pairs with ATG5–ATG12 complexes to induce the autophagosome extension and microtubule-associated protein light chain 3 (LC3) involved in autophagosome extension and maturation [36]. In this study, we found that SNPs increased the BECLIN-1, ATG16L, and LC3-II levels (Figure 5), indicating that SNPs activated the process of autophagosome formation, extension, and maturation. During the process of autophagy induction, LC3 is proteolytically cleaved by ATG4B to generate LC3-I; activated LC3-I is then cleaved by ATG7, ATG3, and ATG5–ATG12–ATG16L complexes to generate LC3-II [37]. LC3-II directly interacts with P62 to deliver ubiquitinated proteins for autophagic degradation [38]. P62, as an autophagic substrate, is widely used as a predictor of autophagic flux [39]. In our study, P62 had no significant difference after treatment with 200 and 400 μg/mL SNPs (Figure 5), indicating that SNPs induced unobstructed autophagosome flux. SNPs were previously reported to induce autophagy activation in different cells, such as murine spermatocytes [22], RAW 264.7 macrophages [23,24], human umbilical vein endothelial cells (HUVECs) [40], human lung epithelial (BEAS-2B) cells [41], colon cancer (HCT-116) cells [42], preosteoblasts (MC3T3-E1) [43], and normal human hepatic (L-02) cells [44]. Herein, we demonstrate that SNPs can activate autophagy. 

In this study, we treated cells with RAP to activate autophagy and with 3-MA and CQ to inhibit autophagy prior to treatment with SNPs. We found that SNP-induced BECLIN-1 and LC3-II levels were further significantly increased and the P62 level was decreased, whereas BECLIN-1 and LC3-II were decreased by 3-MA, and P62 and LC3-II were significantly increased by CQ via the inhibition of autophagic flux (Figure 6). The results are in good agreement with previous studies, where Xi et al. found that RAP increased the BECLIN-1 and LC3-II levels, whereas 3-MA inhibited the BECLIN-1 and LC3-II levels combined with mesoporous spherical SNPs (150 nm) in RAW 264.7 macrophages [24]. Ha et al. found that 3-MA inhibited the LC3-II level, whereas CQ increased the P62 and LC3-II levels combined with mesoporous spherical SNPs (50 nm) in MC3T3-E1 cells [43]. The evidence above shows that SNPs can enhance BECLIN-1-mediated autophagy at the dosages investigated.

To determine the effect of BECLIN-1-mediated SNP-activated autophagy on cell apoptosis in PLCs, SNP-induced cell apoptosis was inhibited by RAP, whereas it was enhanced by 3-MA and CQ (Figure 6). Consistent with a previous study, SNPs (236 nm) activated autophagy while protecting RAW 264.7 macrophages from cell death, and 3-MA combined with SNPs not only increased cell death, but also resulted in cell membrane integrity loss in RAW 264.7 macrophages [23]. Furthermore, we determined the levels of apoptosis-related proteins. We found that SNP-induced cleaved caspase 8, the BAX/BCL-2 ratio, and cleaved caspase 3 levels were significantly decreased by RAP, whereas they were increased by 3-MA and CQ (Figure 6). We knocked down BECLIN-1 by RNA interference to block autophagy. Compared with SNPs alone, the inhibition of autophagy by knockdown of BECLIN-1 significantly decreased the BECLIN-1 and LC3-II levels, whereas it increased the cleaved caspase 8, the BAX/BCL-2 ratio, and cleaved caspase 3 levels (Figure 7). Some recent studies have shown that SNPs induced autophagy and apoptosis [45], but only a few of them elucidated the relationship between autophagy and apoptosis. Our findings indicate that the activation of BECLIN-1-mediated autophagy inhibits SNP-induced cell apoptosis in Leydig cells. 

BECLIN-1, a novel BCL-2 homology (BH)-3 domain-only protein [17], can interact with antiapoptotic protein BCL-2 via its BH3 domain [16]. However, unlike other known BH3-only proteins, BECLIN-1 does not exert a proapoptotic function [46]. In contrast, BECLIN-1 plays an antiapoptotic role in several conditions, such as nutrient deprivation [47], hypoxia [48], and nanoparticle exposure [49]. BCL-2 interacts with the BH3 domain of BECLIN-1, resulting in inhibited autophagy activation, where both autophagy blockage and autophagy gene depletion can cause cell apoptosis under stress stimulation, whereas downregulation of BCL-2 leads to the activation of autophagy [18]. In this study, we found that at the concentrations studied, Stöber SNPs increase the BECLIN-1 level and decrease the BCL-2 level (Figure 5), thus promoting BECLIN-1-mediated critical complex formation in autophagy initiation. Furthermore, we found that BECLIN-1 depletion inhibited autophagy activation via the decrease of the LC3-II level, indicating that BECLIN-1 played an important role in SNP-induced autophagy initiation. It has been reported that SNPs can induce spermatocyte cell apoptosis via caspase 8-mediated cell death pathway [50,51]. In this study, we found that SNPs could increase the cleaved caspase 8 level (Figure 5), indicating caspase 8-mediated cell death cooperated in SNP-induced cell toxicity in the PLCs. The activation of BECLIN-1-mediated autophagy by RAP decreased the cleaved caspase 8 level, while the inhibition of BECLIN-1-mediated autophagy by 3-MA and CQ increased the cleaved caspase 8 level, indicating that cleaved caspase 8 could be regulated by BECLIN-1-mediated autophagy (Figure 6). These findings are consistent with a previous report where the authors demonstrated that cleaved caspase 8 can be degraded by autophagy under conditions associated with cytoprotective autophagy [19]. Liu et al. demonstrated that BECLIN-1-mediated autophagy protects against cadmium-induced activation of apoptosis via the interaction of BECLIN-1 with cleaved caspase 8 to impair its proapoptotic activation [20]. Caspase 8 not only is involved in death receptor-mediated apoptosis, but also cooperates in mitochondria-mediated apoptosis. Caspase 8 and Bid can interact with mature cardiolipin of the mitochondria, resulting in the initiation of early apoptotic signaling [52]. Our study showed that BECLIN-1 depletion increased the cleaved caspase 8 level (Figure 7), further indicating that BECLIN-1-mediated autophagy protects from SNP-induced cell apoptosis via the inhibition of caspase 8 in PLCs. 

Autophagy is a double-edged sword for cell survival and death. SNPs can be taken up by cells via endocytosis and are sequestrated and accumulated by autophagosomes or lysosomes to degrade them. The intensity of the autophagic response is influenced by size, charge, dose, and treatment time of SNPs [41]. Positively charged and larger SNPs (≥100 nm) during higher treatment doses (≥500 μg/mL) and longer time (24 h) can induce autophagy, resulting in autophagy-mediated cell death [41]. Hence, SNPs are able to induce an increase in autophagosome formation and flux and autophagic dysfunction. The LC3-II protein is increased in both categories; however, P62 is only increased in the case of autophagic dysfunction, which is no longer degraded via autophagy [53]. For example, Wang et al. showed that high doses of 60 nm and 16 nm of nonporous spherical SNPs inhibited autophagosome degradation, leading to autophagy dysfunction via detecting the P62 level increase in HepG2 cells and BEAS-2B cells, respectively [54,55]. In this study, we found that 800 μg/mL of SNPs also increased the P62 level, indicating that high doses of SNPs inhibited autophagosome degradation, resulting in autophagy dysfunction in the PLCs (Figure 5), which would be determined in a further study. In summary, SNPs either induced or disrupted autophagy in connection with cell type, particle dose, and treatment time. These doses may or may not be relevant to human exposure depending on the extent and route of exposure.

**Figure 8:**
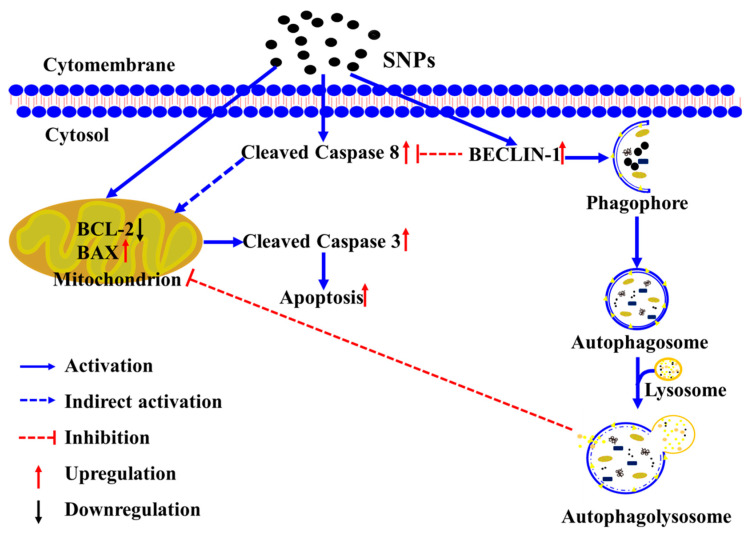
The schematic diagram of the signaling pathway involved in SNP-induced autophagy and apoptosis.

The findings from this study demonstrate that SNPs cause cell apoptosis in Leydig cells, which provides a possible mechanism of SNP-caused testicular toxicity. Furthermore, BECLIN-1-mediated autophagy inhibits SNP-induced cytotoxicity in Leydig cells via the inhibition of the mitochondria-mediated apoptotic pathway and even the death receptor-mediated death signaling, which provides a strategy to relieve or abate SNP-caused testicular toxicity via supplementary autophagy activators. A diagram of the mechanisms involved in SNP-induced cytotoxicity in PLCs summarizes these findings as shown in Figure 8.

## 5. Conclusions

In summary, SNPs are able to activate autophagy via the enhancement of autophagosome formation, extension, and maturation and induce cell apoptosis via the intrinsic apoptotic pathway in Leydig cells. Meanwhile, the activation of autophagy protects Leydig cells from SNP-induced apoptosis via the inhibition of the mitochondria-mediated apoptotic pathway and even the death receptor-mediated death signaling.

## Data Availability

The data presented in this study are available upon reasonable request.
